# Phosphatidylethanolamine positively regulates autophagy and longevity

**DOI:** 10.1038/cdd.2014.219

**Published:** 2015-01-09

**Authors:** P Rockenfeller, M Koska, F Pietrocola, N Minois, O Knittelfelder, V Sica, J Franz, D Carmona-Gutierrez, G Kroemer, F Madeo

**Affiliations:** 1 Institute of Molecular Biosciences, NAWI Graz, University of Graz, Humboldtstr. 50, 8010 Graz, Austria; 2 INSERM U848, Villejuif, Paris, France; 3 Biomedical Sciences Research Complex, University of St Andrews, St Andrews, UK; 4 Metabolomics Platform, Institut Gustave Roussy, Villejuif, Paris, France; 5 Centre de Recherche des Cordeliers, Paris, France; 6 Pôle de Biologie; Hôpital Européen Georges Pompidou, AP – HP, Paris, France; 7 Université Paris Descartes, Sorbonne Paris Cité, Paris, France; 8 BioTechMed-Graz, Humboldtstr. 50, 8010 Graz, Austria

## Abstract

Autophagy is a cellular recycling program that retards ageing by efficiently eliminating damaged and potentially harmful organelles and intracellular protein aggregates. Here, we show that the abundance of phosphatidylethanolamine (PE) positively regulates autophagy. Reduction of intracellular PE levels by knocking out either of the two yeast phosphatidylserine decarboxylases (PSD) accelerated chronological ageing-associated production of reactive oxygen species and death. Conversely, the artificial increase of intracellular PE levels, by provision of its precursor ethanolamine or by overexpression of the PE-generating enzyme Psd1, significantly increased autophagic flux, both in yeast and in mammalian cell culture. Importantly administration of ethanolamine was sufficient to extend the lifespan of yeast (*Saccharomyces cerevisiae*), mammalian cells (U2OS, H4) and flies (*Drosophila melanogaster*). We thus postulate that the availability of PE may constitute a bottleneck for functional autophagy and that organismal life or healthspan could be positively influenced by the consumption of ethanolamine-rich food.

Phosphatidylethanolamine (PE) is a phospholipid found in all living organisms. Together with phosphatidylcholine (PC), phosphatidylserine (PS) and phosphatidylinositol (PI), PE represents the backbone of most biological membranes. PE is the second-most abundant phospholipid in mammalian membranes ranging from 20 to 50%.^1^ In yeast, PE is essential for growth and is generated through four different enzymatic pathways:^2^ PE can be produced by decarboxylation of PS, as a first option at the mitochondrial membrane via phosphatidylserine decarboxylase 1 (Psd1)^3, 4^ or, as a second, option at the Golgi and vacuolar membranes through phosphatidylserine decarboxylase 2 (Psd2).^5^ As a third possibility, PE can be produced from actively retrieved extracellular ethanolamine,^6, 7^ which is cytidine 5'-diphosphate-activated^8^ and then coupled to diacylglycerol to generate PE.^9^ The fourth, scarcely employed PE-generating pathway is based on the lysophospholipid acylation of lyso-PE. Importantly, PE does not spontaneously assemble in bilayers and rather incorporates into curved structures, such as the inverted hexagonal phase.^10^ The physiological function of non-bilayer lipids in membranes is considered to reside in their interaction with membrane proteins via the membrane lateral pressure^10^ and membrane tethering and fusion processes, which are relevant for autophagy.^11^

The term ‘autophagy' describes a degradation process affecting intracellular components (for a review see, 12 13) which as an important cytoprotective mechanism, is closely linked to ageing. Autophagy mainly differs from the proteasomal pathway, the other major cellular degradation mechanism, in two aspects. First, autophagy can degrade large particles or whole organelles and second, the final degradation occurs in the lysosome/vacuole and not at the proteasome. Prior to the actual degradation, the cargo is gathered in autophagic particles, which are surrounded by a characteristic double-membrane. However, the origin of these autophagosomal membranes is still controversial and might actually depend on the mode of autophagy induction.^14, 15^ Among the discussed membrane sources are the Golgi apparatus, the endosplamic reticulum (ER) or the mitochondrion-associated membrane, which is formed at the interface between the ER.^16^ In higher eukaryotes autophagic membranes are enriched in PE with a high degree of unsaturation,^17^ similarly to the PE species found in mitochondria.^14, 18^ Moreover, the pre-autophagosomal structure or phagophore assembly site (PAS), which appears at the very beginning of autophagosome formation, already harbours Atg9, an autophagy-related transmembrane protein that shuttles between mitochondria and the PAS structure in yeast.^19^

Importantly, PE also functions as an anchor to autophagosomal membranes for the autophagy-related protein Atg8 in yeast^20^ and its mammalian orthologue LC3.^21, 22^ This PE anchor is provided to LC3/Atg8 post-translationally in a process called lipidation. First, LC3/Atg8 is carboxy-terminally cleaved by proteases from the Atg4 family.^23, 24^ Subsequently, the remaining C-terminal glycine is coupled to PE in a series of ubiquitination-like reactions involving diverse Atg-proteins.^20, 25, 26, 27^ *In vitro*, Atg8-PE causes hemifusion of vesicles, which argues for its potential role in autophagosomal phagophore expansion.^11, 28^ Consistently, semisynthetic LC3-PE has recently been described to stimulate membrane tethering and fusion.^29^ We thus reasoned that the overall abundance of PE might be critical for PE-lipidation of LC3/Atg8 and could thus regulate autophagosomal membrane formation. Therefore, we tested whether increasing cellular PE levels might have an impact on autophagy and lifespan regulation.

Here, we report that knock-out of *PSD1* or *PSD2* shortens the chronological lifespan of *S. cerevisiae*, whereas PSD1-overexpression enhances the autophagic capacity and increases longevity. Furthermore, external administration of ethanolamine increases endogenous PE levels, enhances autophagic flux and extends the lifespan of yeast, mammalian cells in culture and flies (*Drosophila melanogaster)*.

## Results

### A genetic screen identifies *psd1*Δ and *psd2*Δ as progeroid yeast strains

**Figure 1:**
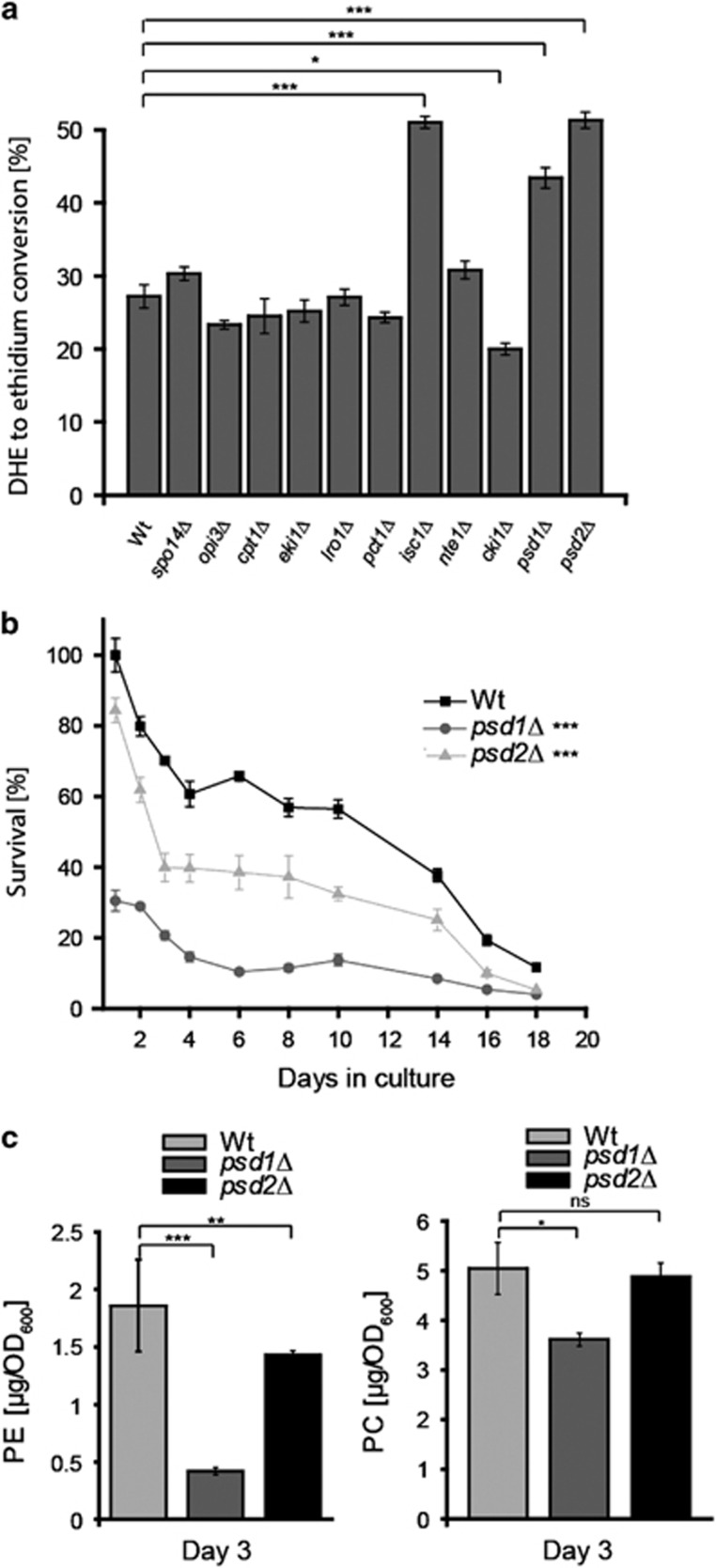
Identification of *psd1*Δ and *psd2*Δ as progeroid yeast strains. (**a**) The ROS-driven conversion of DHE to ethidium was measured at day three of chronological ageing in eleven yeast strains deficient for different enzymes involved in phospholipid synthesis. (**b**) The chronological ageing curve, which is based on the clonogenic survival, is shown for *psd1*Δ and *psd2*Δ in comparison with the wild type. (**c**) Phosphatidylethanolamine (PE) and phosphatidyl choline (PC) were quantified by HPLC-ELSD of lipid extracts from wild-type *psd1*Δ and *psd2*Δ cells at day three of chronological ageing

We designed a screen to identify genes involved in phospholipid anabolism and catabolism that might have an impact on ageing. For this purpose, we performed chronological ageing experiments with a subset of yeast strains deleted for non-essential genes known to be involved in phospholipid metabolism. At day 3 of the chronological ageing experiment, we measured the levels of reactive oxygen species (ROS) by assessing the ROS-driven oxidation of non-fluorescent dihydroethidium (DHE) to fluorescent ethidium by cytofluorometry. This screening procedure led to the identification of three genes whose deletion caused an ageing-dependent raise in ROS generation (Figure 1a): *isc1*Δ, *psd1*Δ and *psd2*Δ. *ISC1* encodes an inositol phosphosphingolipid phospholipase, which produces ceramide. Interestingly, deletion of *ISC1* has been previously reported to decrease chronological lifespan,^30^ thus validating our screen results. *PSD1* and *PSD2* encode phosphatidylserine decarboxylases, which similarly convert PS to PE, and are located in distinct cellular organelles (see Introduction). As these two enzymes have not been associated with yeast aging, we decided to focus our study on these enzymes and their products.

We confirmed the premature ageing phenotype of the *PSD1* and *PSD2* knock outs by clonogenic survival plating (Figure 1b) and assessed the PE- and PC-abundance at day 3 of ageing by HPLC-assisted analyses of lipid extracts (Figure 1c). *PSD1* deletion had a more pronounced inhibitor effect on both PE synthesis and yeast ageing. This is consistent with a previous report underscoring that, at least in standard culture conditions, Psd1 is the predominant phosphatidylserine decarboxylase, the abrogation of which disturbs homeostasis of PE and a number of phospholipid species including PS and PI which can be overcome by the administration of ethanolamine.^31^

As previous works have also shown that *PSD1* deletion has a strong impact on mitochondrial function,^32, 33^ we examined to which extent the reduction in survival upon chronological ageing might be a consequence of general mitochondrial dysfunction or specific to the deletion of PSD activity. We thus generated *psd1Δ* and *psd2Δ* strains, which are additionally rendered defective for respiration owing to loss of mitochondrial DNA (Rho^0^). These Rho^0^ strains showed reduced survival rates compared with their Rho^+^ counterparts starting from day 2 of chronological ageing (Supplementary Figure S1A). Still, deletion of mitochondrial DNA exacerbates the premature ageing phenotype of *psd1*Δ indicating that mitochondria are at least partially functional in *psd1*Δ.

In addition, experiments using growth media containing the non-fermentable carbon source glycerol (Supplementary Figure S1B,C, Supplementary Results) suggest that lack of Psd1 can be compensated over time, presumably by Psd2 activity, providing enough PE to establish functional mitochondria with a sufficient respiratory capacity. We summarise that although during early stages of chronological ageing mitochondrial defects are occurring in *psd1*Δ, at later time points other effects of *PSD1* deletion such as autophagy may become prominent.

### PSD1 overexpression extends yeast chronological lifespan and increases autophagy

**Figure 2:**
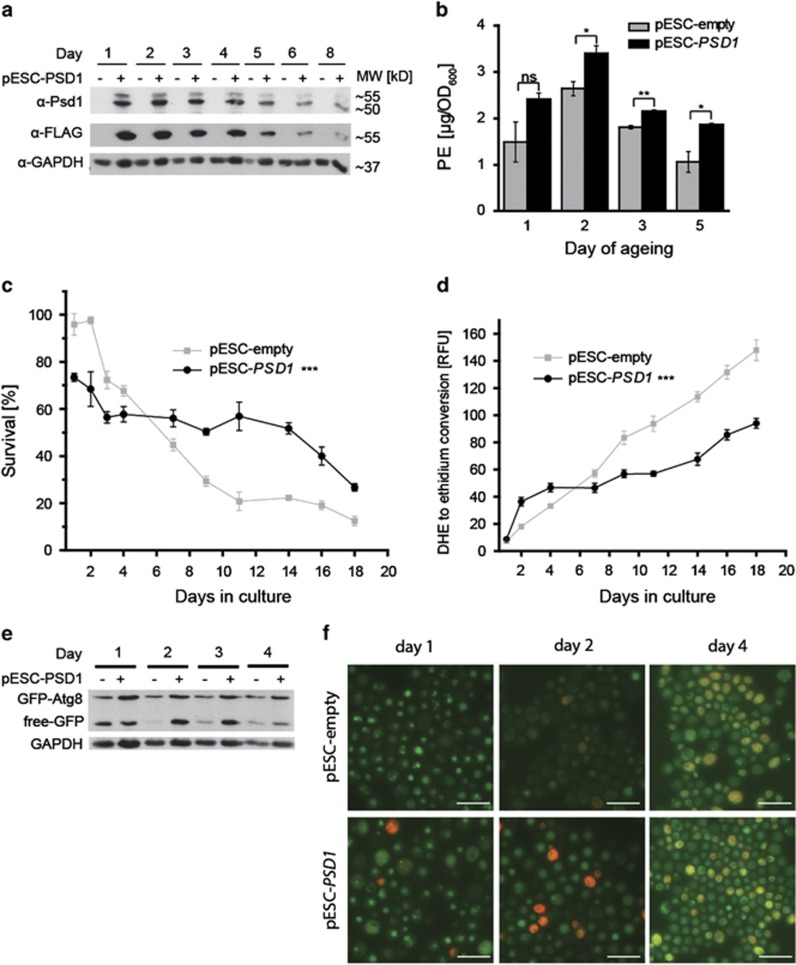
PSD1 overexpression extends chronological lifespan of wild-type yeast and increases autophagy. (**a**) Representative immunoblot monitoring Psd1 overexpression levels in conditions of chronological ageing. Full length Psd1-flag was detected by anti-Psd1 and anti-flag antibody at ~55 kDa, whereas the cleaved protein (active form^57^) was only detected by the specific Psd1 antibody at ~50 kDa. GAPDH abundance was assayed as loading control. (**b**) HPLC-ELSD-assisted quantification of total cellular phosphatidyl ethanolamine (PE) levels. (**c**) Clonogenic survival (**d**) ROS-driven conversion of DHE to ethidium in yeast chronological ageing is shown in control cells and in cells that overexpress *PSD1*. (**e**) Detection of GFP-Atg8 and GFP by immunoblotting at different days of ageing of cells that normally or overexpress *PSD1*. The band corresponding to free GFP (liberated from GFP-Atg8) is regarded as a measure of autophagy flux. (**f**) Representative GFP-Atg8 microscopy images of *PSD1*-overexpressing cells at day 1, 2 and 4 of chronological ageing. Scale bar=10 *μ*m

As the deletion of Psd1 activity decreased yeast lifespan, we wondered whether *PSD1* overexpression in wild-type yeast would have the opposite effect. First, we confirmed functionality of the *PSD1* overexpression (Figure 2a, Supplementary Results, Supplementary Figure S2A, B). Quantification of total cellular PE levels revealed that PE was significantly higher in cells overexpressing *PSD1* (Figure 2b), where PC levels remained unchanged at least on days 1 to 3 (Supplementary Figure S2C). Next we demonstrated that indeed, *PSD1* overexpression increased the clonogenic survival (Figure 2c) and decreased ROS accumulation (Figure 2d) during chronological ageing. Interestingly, this increased PE-abundance correlated with an upregulation of autophagy, as detected by GFP-Atg8 immuno blotting (Figure 2e, Supplementary Figure S2D). The free GFP band, which indicates vacuolar degradation of GFP-Atg8, represents a measure for successful autophagy execution.^34, 35^ *PSD1* overexpression led to an increase in the intensity of the band corresponding to free GFP at days 2 and 3 of ageing relative to control cells transformed with vector only (Figure 2e). In addition, we evaluated microscopically whether vacuoles were GFP-positive, which is indicative for completed autophagy. Indeed, *PSD1* overexpression increased the amount of GFP-positive vacuoles at days 2 and 4 (Figure 2f). Measurement of alkaline phosphatase (ALP) activity (Supplementary Figure S2E), which is known to increase with autophagy in a genetically engineered yeast strain,^36, 37^ confirmed that *PSD1* overexpression increases autophagy flux. Altogether, these data suggest that a rise in intracellular PE mediated by *PSD1* overexpression leads to chronological lifespan extension through increased autophagy.

### External supply of ethanolamine enhances the endogenous PE pool, induces autophagy and extends yeast lifespan

**Figure 3:**
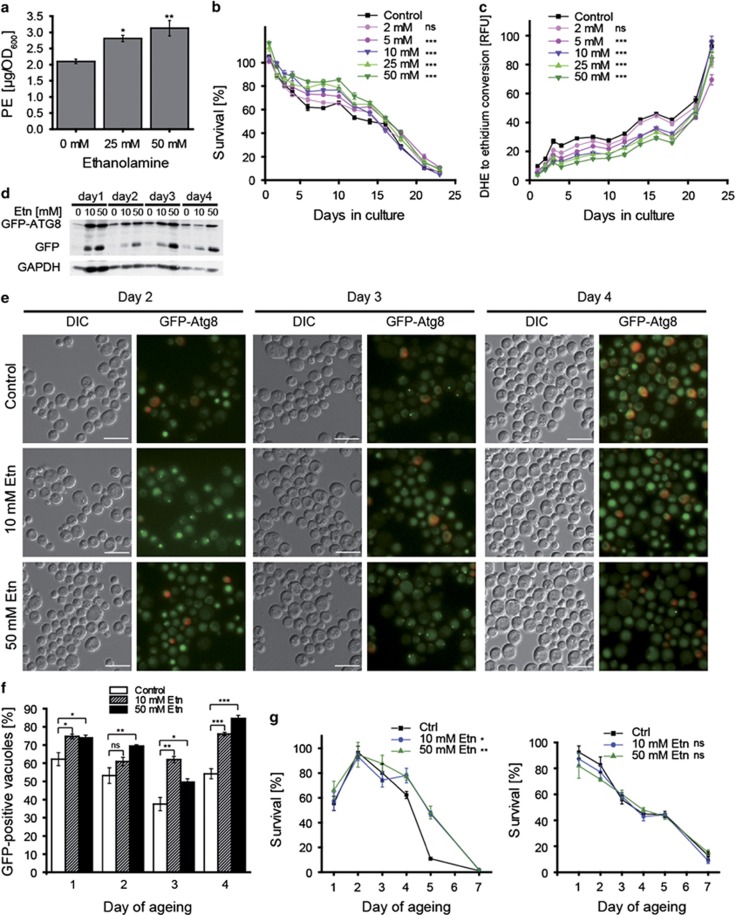
Administration of ethanolamine enhances the endogenous PE pool, induces autophagy and extends yeast lifespan. (**a**) HPLC-ELSD-assisted measurement of total cellular PE levels. (**b**) Clonogenic survival plating and (**c**) DHE to ethidium conversion throughout yeast chronological ageing upon addition of the indicated doses of ethanolamine. (**d**) Representative GFP-Atg8 immunoblot. The band corresponding to free GFP (liberated from GFP-Atg8) is considered as a measure of autophagic flux. (**e**) Representative microphotographs of GFP-Atg8-expressing cells under conditions of ethanolamine (Etn) administration. Scale bar=5*μ*m. (**f**) GFP-Atg8 positive vacuoles were quantified in live (PI-negative) cells. (**g**) Chronological ageing of BY4742 wild type (left panel) and atg7Δ (right panel) with 0, 10 and 50 mM ethanolamine treatment

We next evaluated the hypothesis that external supply of ethanolamine might additionally increase the intracellular PE pool by conversion into PE through the Kennedy pathway.^2^ Indeed, administration of ethanolamine to yeast culture stimulated a substantial augmentation in intracellular PE levels (Figure 3a). Ethanolamine also caused a concentration-dependent increase in yeast cell survival (Figure 3b), coupled to a decrease in ROS production (Figure 3c) during chronological ageing. Moreover, ethanolamine enhanced autophagic flux as documented by GFP-Atg8 immunoblotting (Figure 3d, Supplementary Figure S3A), microscopic assessment of the subcellular localisation of GFP-Atg8 (Figures 3e and f) and measurement of ALP activity (Supplementary Figure S3B). It should be noted that the administration of ethanolamine lead to a small, but significant change in the pH of culture media (Supplementary Figure S3C). However, this change should not be relevant for the observed positive effects on chronological ageing as the pH decreased, which is known to negatively impact on chronological ageing.^38^ We performed additional control experiments to exclude the possibility that ethanolamine might exert anti-ageing effects as a nitrogen source or owing to its caloric value. However, administration of neither ammonium sulphate (nitrogen control) nor ethanol (calorie control) did mimic the effects of ethanolamine (Supplementary Figure S3D). We also provide evidence that inhibition of ethanolamine driven PE synthesis via the Kennedy pathway by *ECT1*Δ *PCT1*Δ double deletion abolishes ethanolamine-mediated lifespan extension (Supplementary Figure S3E). Ect1 is the cytidyltransferase preferably activating phosphoethanolamine to be further converted into PE, whereas Pct1 is the cytidyltransferase preferably utilising phosphocholine as substrate to synthesise PC. As the two proteins are redundant in their substrate specificity, only the double knock out entirely inhibits PE synthesis by the Kennedy pathway. In addition, inhibition of autophagy by *ATG7* deletion abrogated the beneficial effects of ethanolamine treatment on the chronological lifespan (Figure 3g) or at least partially reduced its positive effects, depending on the genetic strain background (Supplementary Figure S3F). Our findings thus support the notion that intracellular PE levels may affect the lifespan through regulation of autophagy.

### Ethanolamine administration induces autophagy and reduces chronological senescence in mammalian cells

**Figure 4:**
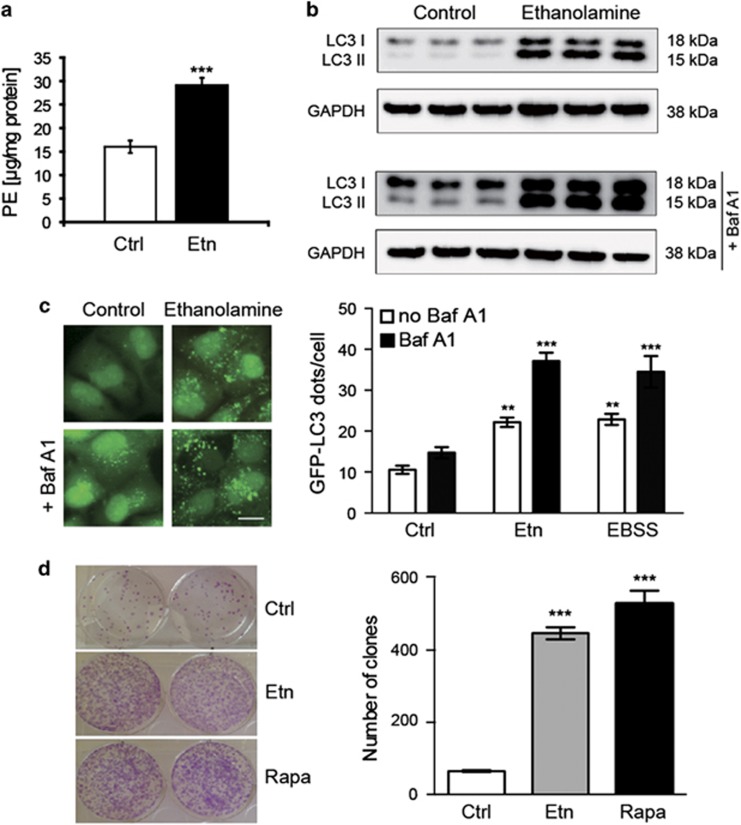
Administration of ethanolamine induces autophagy and reduces chronological senescence in mammalian cells. (**a**) HPLC-ELSD-assisted measurement of U2OS total cellular PE levels with and without 10 mM ethanolamine (Etn) treatment. (**b**, **c**) Effect of ethanolamine administration on autophagy induction. Wild-type human osteosarcoma U2OS cells (**b**) and their GFP-LC3 expressing counterparts (**c**) were either left untreated or treated with 10 mM ethanolamine. Thereafter, cells were processed for the immunochemical detection of LC3 lipidation (**b**) and for fluorescence microscopic quantification of GFP-LC3 positive dots (**c**). Administration of ethanolamine resulted in increased autophagy flux, as measured by LC3 lipidation in the absence or in the presence of bafilomycin A1 (bafA1). GAPDH levels were detected to ensure equal loading. Representative immunoblots are depicted in (**b**). Autophagy induction by ethanolamine treatment was confirmed by microscopic detection of GFP-LC3^+^ puncta in U2OS cells stably expressing GFP-LC3, in the absence or in the presence of bafilomycin A1 (**c**). Nutrient deprivation (EBSS) was used as a positive control of autophagy stimulation. (**d**) Ethanolamine reduces yeast-like chronological senescence and increases replicative viability in mammalian cells. Highly confluent U2OS cells were left untreated or stimulated with 10 mM ethanolamine or 1 *μ*M rapamycin (Rapa, positive control) for one week

Addition of ethanolamine to human osteosarcoma (U2OS) and neuroblastoma (H4) cell cultures resulted in increased PE levels (Figure 4a, Supplementary Figure S4A), whereas the endogenous PC levels remained unaffected (Supplementary Figure S4B, C). Furthermore, the autophagic flux, as measured by LC3 lipidation in the absence and presence of the autophagy inhibitor bafilomycin A1 was also increased as a response to ethanolamine treatment (Figure 4b). The bands corresponding to the lipidated fraction of LC3 (LC3II) showed an increased intensity upon ethanolamine treatment as compared with untreated cells or cells exposed to bafilomycin A1 alone. These data were confirmed by fluorescence microscopic detection of autophagic puncta in U2OS cells stably expressing the autophagic marker GFP-LC3 (Figure 4c). The number of GFP-LC3-positive puncta significantly increased in response to ethanolamine, both in the absence and presence of bafilomycin A1. Of note, the level of ethanolamine-mediated autophagy was equivalent to that observed upon nutrient deprivation (namely cultured in Earl's balanced salt solution), which was used as a positive control of autophagy stimulation. Autophagy induction by ethanolamine was neither associated with a decrease in mTORC1 activity, as measured by the phosphorylation of p70^s6k^, nor with an activation of AMPK (Supplementary Figure S4D). All these experiments were repeated in a H4 neuroblastoma cell line stably expressing GFP-LCD, yielding similar results (data not shown). Hence, ethanolamine can stimulate autophagy in a variety of human cell lines. To assess cell survival, we finally performed a yeast-like chronological senescence assay, where highly confluent U2OS cells were left untreated or stimulated with 10 mM ethanolamine for 1 week. Remarkably, ethanolamine treatment largely prevented the senescence-induced decrease of clonogenic cell survival (Figure 4d). In fact, ethanolamine was almost as effective as the potent autophagy stimulator rapamycin in improving human cell survival in over-confluent conditions.

### Ethanolamine increases lifespan in *Drosophila*

**Figure 5:**
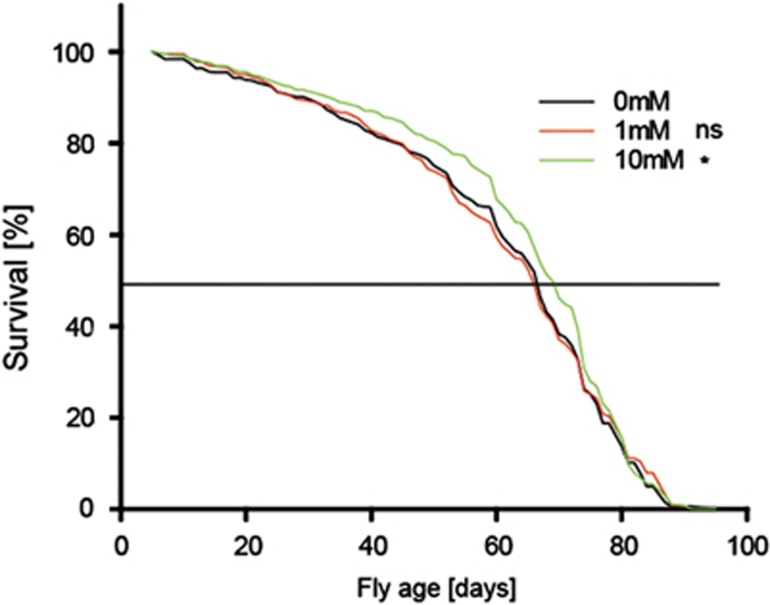
Ethanolamine administration enhances the lifespan of *Drosophila melanogaster*. Pooled data of five independent female fly lifespan measurements. The mean lifespan of the control population is 60.98±0.96, 60.44±0.98 for flies treated with 1 mM ethanolamine and 64.05±0.90 days for 10 mM ethanolamine-treated flies. Wilcoxon comparison of 1 mM treated flies with control flies revealed a *χ*^2^ of 0.2 on 1 degree of freedom and *P*=0.688, whereas a significant lifespan increase was assessed comparing 10 mM with untreated flies with a *χ*^2^ of 5.5 on 1 degree of freedom and *P*=0.0192. See Supplementary Figure S5 for individual lifespan analyses

We finally evaluated the effect of ethanolamine on the fruit fly, *Drosophila melanogaster*. We supplied flies with ethanolamine and monitored their lifespan in five independent analyses as compared with untreated controls (Supplementary Figure S5). The pooled results revealed that the mean lifespan of flies treated with 10 mM ethanolamine is significantly extended by ~5% (Figure 5).

## Discussion

Autophagy is regarded as one of the major cytoprotective mechanisms during ageing,^12^ and thus is a crucial process to counteract age-associated pathologies. Age-associated neurodegenerative disorders including Alzheimeŕs and Parkinsońs disease may be postponed or attenuated by chronic induction of autophagy,^12, 39^ and there is substantial evidence that genetic or pharmacological induction of autophagy can increase the healthspan and lifespan of multiple model organisms including yeast, worms, flies and mice.^40^ These findings have spurred the interest in identifying novel, non-toxic pharmacological inducers of autophagy. So far, several agents have been shown to induce autophagy and increase lifespan across several species,^40^ namely rapamycin,^27, 40^ resveratrol^40, 41, 42^ and spermidine.^43^ The present results suggest that ethanolamine might be yet another potent autophagy inducer that promotes longevity. Like rapamycin, resveratrol and spermidine, ethanolamine is a naturally occurring component that is endowed with a favourable toxicity profile (note that the oral LD50 in rats is 1.72 g/kg) when compared with rapamycin and resveratrol.

Our study provides evidence that ethanolamine-mediated autophagy induction correlates with enhanced longevity in yeast and mammalian cell culture. This is in line with a previous study in yeast demonstrating that PE is a limiting factor for autophagy in a carboxypeptidase Y-defective background.^44^ Our results demonstrate that these observations are applicable to a wild-type scenario in yeast too and can be extended to mammalian cell cultures. Still, future experiments will need to clarify if ethanolamine-induced autophagy is beneficial to higher organisms. We could indeed observe a significant increase in the mean lifespan of flies upon supplementation with ethanolamine, but whether this is because of autophagy must be tackled in the follow-up studies.

Besides the pharmacological application of ethanolamine as an anti-ageing agent, our results underline the importance of this molecule for autophagy regulation. *In vivo*, phosphatidylserine decarboxylase plays a major role in the supply of PE, which is a major component of mitochondrial membranes in yeast^45^ and mammals.^1^ It is thus not surprising that the abrogation of yeast *PSD1* or mammalian *PISD* affects mitochondrial dynamics such as mitochondrial fusion processes^32, 33, 46^ and reduces yeast lifespan (this study). Interestingly, in psd1Δ Rho^0^ the survival rates at day 1 exceed those of the Rho^+^ counterpart, which suggests that a mitochondrial signal might be involved as a cell death trigger. Similarly, the phenotype of *PISD* homozygous knock-out mice is so severe that embryos die at day 8 to 10 of embryonic development.^46^ In another yeast study Psd2 has been described as being important for PE synthesis and autophagy under conditions of cadmium stress.^47^ This is interesting as it generally underlines the importance of PE-abundance for functional autophagy. However, in most of our experiments we observed stronger effects relating to Psd1 function. We showed that *PSD1* overexpression induced autophagy and increased the yeast lifespan. Genetic abrogation of autophagy-related factors in the overexpression setting would in addition strengthen the hypothesis that autophagy was crucial for lifespan extension, which will be tackled in a follow-up study. Our yeast and mammalian data indicate that ethanolamine treatment triggers autophagy through a phylogenetically conserved pathway. Moreover, at least in mammalian cells autophagy induction by ethanolamine was neither associated with a decrease in mTORC1 activity, as measured by the phosphorylation of p70^s6k^, nor with an activation of AMPK (Supplementary Figure S4). Although we cannot exclude that alternative regulation processes are involved, it is possible that the availability of PE alone is sufficient to signal autophagy induction.

Of note, lipid determination during chronological ageing, might be diffused to some extent, as lipid extracts are not solely derived from living, but also from dead cells, which might still reside lipolytic activity and thus compromise the results of lipid profiling. However, this should not only affect PE content but similarly other phospholipid classes such as PC, PI or PS. Using the same lipid extracts as for PE-detection, PC-measurement did not reveal significant change for *psd2Δ*, but slightly decreased PC levels in psd1Δ compared with wild type (Figure 1c). Although PC is only reduced by 28% comparing *psd1Δ* to wild type on day 3 of chronological ageing, the PE reduction measures 77% (Figure 1c). Thus, we rather interpret the PE reduction in *psd1Δ* and *psd2Δ* as a predominant consequence of their primary loss of enzymatic function and not as a result of the dead cell population.

One suitable marker of autophagy is the PE-lipidation of LC3/Atg8,^20^ as we use it in this study. However, PE-lipidated LC3 has also been shown to localise to lipid droplets and to contribute to their formation.^48, 49^ The LC3 conjugation system thus seems to be involved in lipid metabolism. In turn, autophagy has also been shown to be directly involved in lipid turnover.^50^ These studies show that lipid metabolism/storage and autophagy share some functional features. It is thus tempting to speculate that PE may act as a crucial molecular (and regulatory) link between autophagy and lipid metabolism.

Altogether, these results have identified ethanolamine as an autophagy-inducing stimulus that will be relevant for future autophagy, lipid and ageing research. Given its anti-ageing potential, ethanolamine might even emerge as a new therapeutic agent in the treatment of age-associated human diseases.

## Materials and Methods

### Yeast strains and growth conditions

All experiments were carried out in the BY4741 (*MATa his3*Δ*1 leu2*Δ*0 met15*Δ*0 ura3*Δ*0*) strain background except for the experiments shown in Figures 3g and h, where BY4742 (*MATα his3*Δ*1 leu2*Δ*0 lys2*Δ*0 ura3*Δ*0*) was used. Single deletion strains were obtained from the EUROSCARF knock-out collection (see Supplementary Table S1 for a complete list of strains). Strains were grown in SC medium containing 0.17% yeast nitrogen base (Difco, BD, Franklin Lakes, NJ, USA), 0.5% (NH_4_)_2_SO_4_ and 30 mg/l of all amino acids (except 80 mg/l histidine and 200 mg/l leucine), 30 mg/l adenine, and 320 mg/l uracil with 2% glucose as carbon source for SCD medium or 2% galactose for SCG medium, respectively. All yeast cultures were inoculated from a stationary overnight culture to an OD of 0.1 at 600 nm (OD_600_=0.1) and then grown at 28 °C and 145 r.p.m. shaking for the indicated time periods. For induction of the *Gal10* promoter to drive *PSD1* expression, pESC-PSD1 containing cells were shifted to SCG medium at an OD_600_=0.35. Ethanolamine chloride from Sigma (St. Louis, MO, USA, E6133) was administered to the cultures from a 1 M stock in ddH_2_O or from a 5 M stock for the 50 mM final concentration.

### Cloning and molecular biology

*PSD1* was cloned into the multiple cloning site 2 of the plasmid pESC His (Agilent Technologies, Santa Clara, CA, USA) by homologous recombination in yeast using the forward primer 5′-TTCGAATTCAACCCTCACTAAAGGGCGGCC_ATGTCAATTATGCCAGTTAAGAAC-3′ and as backward primer 5′-GATCTTATCGTCGTCATCCTTGTAATCCAT_TTTTAAATCATTCTTTCCAATTATGCCTAATTTC-3′.

### Survival plating and ROS determination

For survival plating, the cell concentrations of culture dilutions were determined with a CASY cell counter (Roche Diagnostics, Mannheim, Germany) and aliquots containing 500 cells were plated on YPD plates. The number of colonies formed was determined after 2 days at 28 °C. For dihydroethidium staining, 5 × 10^6^ cells were harvested by centrifugation, resuspended in 250 *μ*l of 2.5 *μ*g/ml DHE in PBS and incubated in the dark for 5 min. Relative fluorescence units were determined using a Tecan GeniusPRO fluorescence reader (Tecan Group, Maennedorf, Switzerland) and then normalised to OD_600_. For ROS analysis on the basis of individual cells, flow cytometry was used to count the positive cells.

### Yeast autophagy measurement

Autophagy was measured by monitoring the cytosol to vacuole translocation of Atg8 using fluorescence microscopy or immunoblotting (GFP liberation assay) of cells/cell extracts from strains carrying a GFP-Atg8 fusion protein^34, 35^ expressed under its endogenous promoter and at its natural chromosomal locus. Quantification of micrographs was performed from blinded pictures with 150–300 cells per micrograph and replicate. Autophagic cells were defined as cells exhibiting clear vacuolar GFP fluorescence and expressed as fraction of viable (PI-negative) cells. Immunoblotting followed standard procedures using anti-GFP (Roche Diagnostics, #11814460001), anti-Psd1 or anti-glyceraldehyde-3-phosphate dehydrogenase (GAPDH) antibodies (both gifts from Dr. Guünther Daum).

### Yeast lipid extraction

Total lipids were extracted from exponentially growing yeast cultures at indicated days of ageing with chloroform/methanol 2:1 (v/v) according to Folch *et al.*^51^ The organic phase was dried under a stream of nitrogen and dissolved in 500 *μ*l of chloroform/methanol (2:1, v/v).

### Lipid analysis

PE was quantified by a normal phase HPLC—evaporative light scattering detector (ELSD) method as described in Guerfal *et al.*^52^ (the method is accepted for publication in Cold Spring Harbor Protocols: ‘Analyzing and understanding lipids of yeast: a challenging endeavour'). In brief, the chromatographic separation of lipids was achieved on a Betasil Diol column (100 × 4.6 mm, particle size 5 *μ*m, Thermo Fisher Scientific Inc., Waltham, MA, USA) with a ternary gradient (modified from 53 and described in detail in Cold Spring Harbor Protocols). PE 34:1 standard (Avanti Polar Lipids, Inc., Alabaster, AL, USA) was prepared as 1 mg/ml stock solution in chloroform/methanol 2:1 (v/v). Calibration curves (triplicates) were measured from 2.7 to 350 *μ*g/ml, 10 *μ*l sample was injected for each measurement.

### Cell culture

#### Chemicals, cell lines and culture conditions

Unless otherwise specified, chemicals were purchased from Sigma-Aldrich (St. Louis, MO, USA), culture media and supplements for cell culture from Gibco-Invitrogen (Carlsbad, CA, USA) and plasticware from Corning (Corning, NY, USA). Human osteosarcoma U2OS cells, their GFP-LC3-expressing derivatives, human neuroblastoma H4 GFP-LC3 (gift from Y. Juan) cells were cultured in DMEM medium containing 10% foetal bovine serum, 100 mg/l sodium pyruvate, 10 mM HEPES buffer, 100 units/ml penicillin G sodium and 100 *μ*g/ml streptomycin sulphate (37 °C, 5% CO_2_). Lipid extractions were performed after 12 h of 10 mM ethanolamine (Sigma, E9508) treatment. For autophagy induction, cells were treated for 12 h with 10 mM ethanolamine or incubated in absence of nutrients. For the yeast-like chronological senescence assay, cells were treated for 1 week with 10 mM ethanolamine or 1 *μ*M rapamycin (R&D Systems, Minneapolis, MN, USA).

#### Lipid extraction from U2OS and H4 cells

Cells were harvested in PBS pH 7.4 and pelleted before shock freezing in liquid nitrogen. Cell disruption was performed by sonication in PBS pH 7.4. Protein concentration was determined using the Bio-Rad protein assay kit (Bio-Rad Laboratories, Hercules, CA, USA) and the results were used for normalisation after HPLC measurement. The raw extracts were extracted with chloroform/methanol (2:1) after Folch.^51^ The organic phase was collected and combined with the organic phase obtained from the re-extraction of the aqueous phase. Organic solvents were evaporated under a stream of nitrogen and the lipids were dissolved in chloroform/ methanol (2:1). Lipid analysis was performed as described above.

### Immunoblotting

For immunoblotting, 25 *μ*g of proteins were separated on 4–12% bis-tris acrylamide (Thermo Fisher Scientific Inc.) and electrotransferred to Immobilon membranes (Merck Millipore, Darmstadt, Germany). Membranes were then sliced horizontally in different parts according to the molecular weight of the protein of interest to allow simultaneous detection of different antigens within the same experiment.^54, 55^ Unspecific binding sites were saturated by incubating membranes for 1 h in 0.05% Tween 20 (v:v in TBS) supplemented with 5% non-fat powdered milk (w:v in TBS), followed by an overnight incubation with primary antibodies specific for LC3B, phospho-AMPK (Thr172), AMPK, phospho-ribosomal protein S6 kinase (Thr421/Ser424), ribosomal protein S6 kinase (Cell Signalling Technology Inc., Danvers, MA, USA). Development was performed with appropriate horseradish peroxidase (HRP)-labelled secondary antibodies (Southern Biotech, Birmingham, AL, USA) plus the SuperSignal West Pico chemoluminescent substrate (Thermo Fisher Scientific Inc.). An anti-glyceraldehyde-3-phosphate dehydrogenase antibody (Chemicon International Inc., Temecula, CA, USA) was used to control equal loading of lanes.

### Automated microscopy

U2OS or H4 cells stably expressing GFP-LC3 were seeded in 96-well imaging plates (BD Falcon, Sparks, USA) 24 h before stimulation. Cells were treated with the indicated agents for 4 h. Subsequently, cells were fixed with 4% PFA and counterstained with 10 *μ*M Hoechst 33342. Images were acquired using a BD pathway 855 automated microscope (BD Imaging Systems, San José, USA) equipped with a 40X objective (Olympus, Center Valley, USA) coupled to a robotised Twister II plate handler (Caliper Life Sciences, Hopkinton, USA). Images were analyzed for the presence of GFP-LC3 puncta in the cytoplasm by means of the BD Attovision software (BD Imaging Systems). Cell surfaces were segmented and divided into cytoplasmic and nuclear regions according to manufacturer standard proceedings. RB 2x2 and Marr–Hildreth algorithms were used to recognize cytoplasmic GFP-LC3 positive dots.

### Yeast-like chronological senescence assay

The assay was performed as described in Leontieva *et al.*^56^ Briefly, 80000 cells were seeded into 96-well plates and left untreated or treated with 10 mM ethanolamine or 1 *μ*M rapamycin. After 8 days, dead cells and conditioning media were removed, cells were trypsinized and a 10% aliquot was plated in fresh medium-filled six-well plates. After 1 week, clones were marked trough crystal violet staining and counted.

### Drosophila lifespan experiments

Female flies from an isogenised w^1118^ strain were used. They were kept in a 25 °C, 70% humidity, 12 h light/12 h dark incubator on a standard cornmeal-sugar-yeast diet. Flies were collected at emergence and 20 females were kept per vial with an average of 87 flies per group and replicate. The food was changed twice a week and supplemented or not with ethanolamine hydrochloride (Sigma, E6133) at 1 mM or 10 mM final concentrations. The number of dead flies were recorded every weekday until all flies were dead. Five independent replicates were performed. The independent replicates, as well as the pooled data from all replicates were analysed by a Wilcoxon survival analysis test in R (script available on request).

### Statistical analysis

Statistical analyses were calculated in Origin8. For assessment of significance one-way ANOVA followed by Bonferroni *post hoc* test was performed, except for Figure 1b, Figures 2a and b and Figures 3b, c, g and h which were processed using a two-factor ANOVA with strain and time as independent factors. Data in Figure 5 were assessed for significant difference by Wilcoxon analysis. Error bars indicate standard error of the mean (SEM) and asterisks in the figures indicate significant differences, *P<0.05, **P<0.01, ***P<0.001.

## Abbreviations

DHE: Dihydroethidium
ER: endoplasmic reticulum
PE: phosphatidylethanolamine
PC: phosphatidylcholine
PI: phosphatidylinositol
PS: phosphatidylserine
Psd/Pisd: phosphatidylserine decarboxylase
PAS: pre-autophagosomal structure/phagophore assembly site
ROS: reactive oxygen species
PI: propidium iodide
