# Supplementary material for: Phosphatidylethanolamine positively regulates autophagy and longevity
Source: Cell Death Differ. 2015 Jan 9;22(3):499–508. doi: 10.1038/cdd.2014.219 (PMC4326582; doi:10.1038/cdd.2014.219)
Supplement: Supplementary Material [file cdd2014219x1.doc]

Supplemental Material

Supplemental Results

We further performed chronological ageing experiments using the non-fermentable carbon source glycerol. A previous work (Birner Gruenberger et al. Mol Biol Cell. 2001) has shown that *PSD1* deleted yeast shows a petite phenotype and does not grow on media containing glycerol as carbon source. However, we find that the growth rate is only reduced and that after ten days the cell density equals those of the wildtype. This makes the analysis of the chronological ageing difficult since the actual ageing process only starts after the cells have reached the stationary phase. However, it suggests that lack of Psd1 can be compensated over time, presumably by Psd2 activity, providing enough PE to establish functional mitochondria with a sufficient respiratory capacity.

In order to confirm that the FLAG-tagged Psd1-fusion protein is functional (it must undergo carboxy-terminal cleavage to be activated (1)) we performed immunoblotting using both a FLAG-directed and a specific Psd1-directed antibody (Fig. 2A, Fig. S2A, B). The strongest band of specific Psd1 detection corresponds to the active form (~50 kD), while the band at ~55 kD identifies the uncleaved Psd1-FLAG fusion protein, which is also recognised by the FLAG-directed immunodetection. Psd1 levels were stable for the first three days of chronological ageing, then gradually diminished until day 8 (Fig. 2A). Psd1 expression in the control cells was only detectable at longer illumination times (Fig. S2A, right panel), as it was far beyond overexpression levels. We also analysed the endogenous Psd1 expression of wildtype cells in a separate chronological ageing and immunoblot (Fig. S2F), confirming basic expression until day 8, which gradually decreases with time.

Supplemental Figure Legends


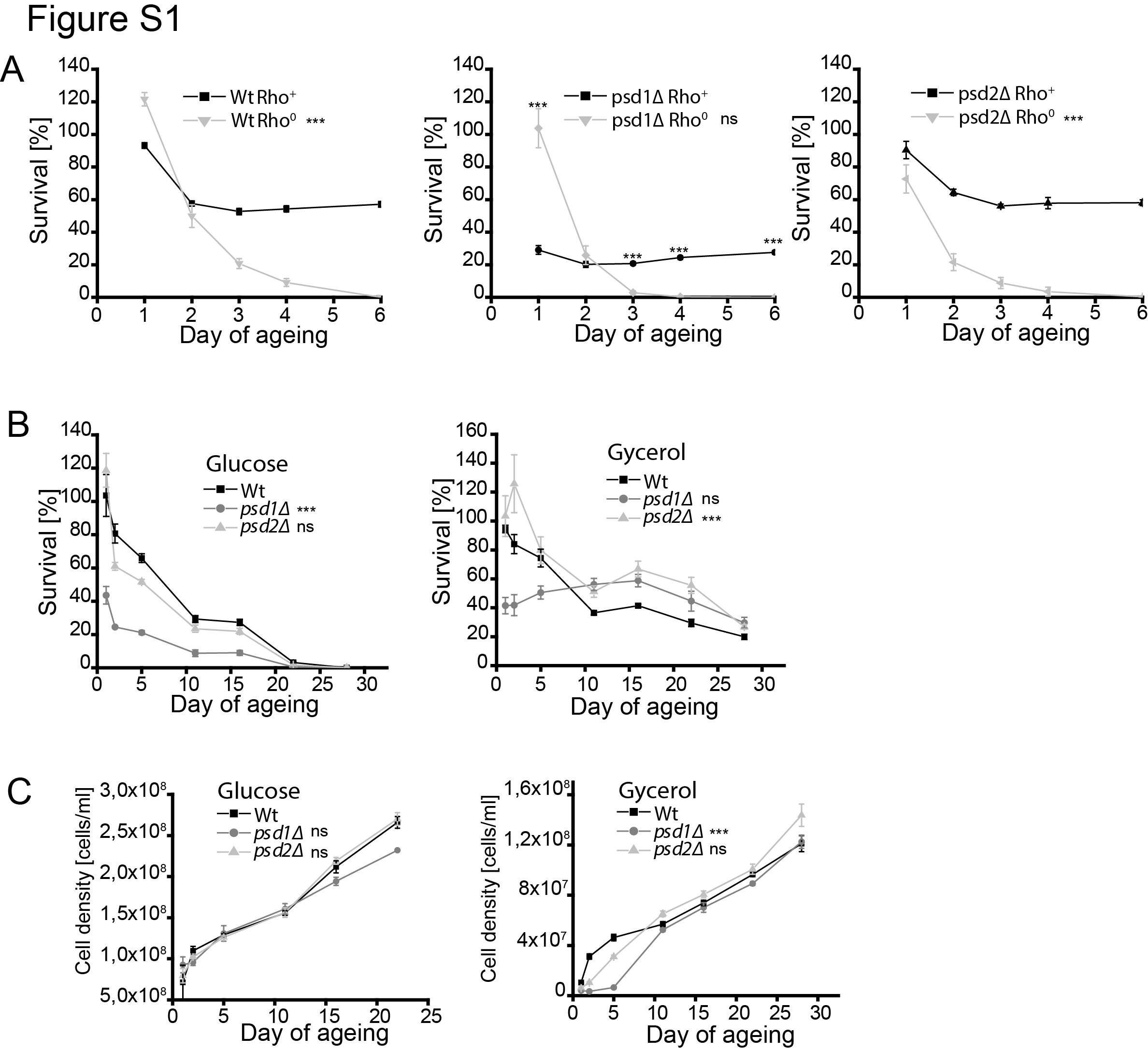


Figure S1. Additional figure related to Fig. 1. (A) Chronological lifespan based on clonogenic survival comparing Rho^+^ to Rho^0^ cells for widtype (left panel), *psd1∆* (middle panel) and *psd2∆* (right panel). (B, C) Chronological lifespan based on clonogenic survival (B) and growth curve (C) comparing widtype, *psd1∆* and *psd2∆* on glucose medium (left panels) and on glycerol medium (right panels).


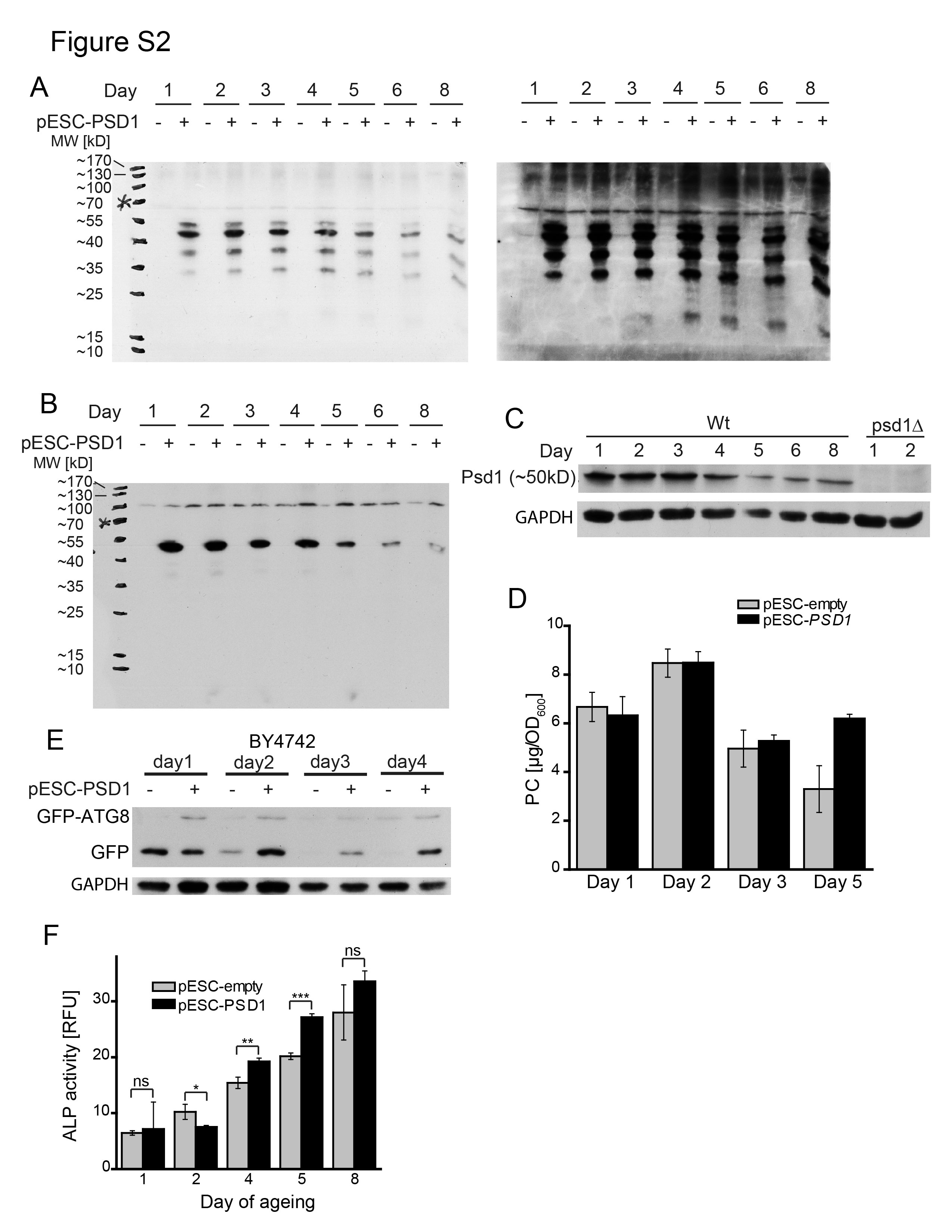


Figure S2. Additional figure related to the *PSD1* overexpression data from Fig. 2. (A, B) Full immunoblot monitoring *PSD1* overexpression during chronological ageing decorated with a specific anti-Psd1 antibody (A) at two different illumination times (left: 10 s, right: 2 min) and anti-FLAG antibody (B). (C) HPLC-ELSD-assisted quantification of total cellular phosphatidylcholine (PC) levels. (D) GFP-ATG8 immuno blot at different days of yeast ageing from BY4742 with and without *PSD1* overexpression. (E) Measurement of alkaline phosphatase activity as a measure of autophagy in yeast with *PSD1* overexpression. (F) Immunoblot showing wildtype *PSD1* expression during ageing using the specific Psd1-antibody and anti-GAPDH antibody as loading control.


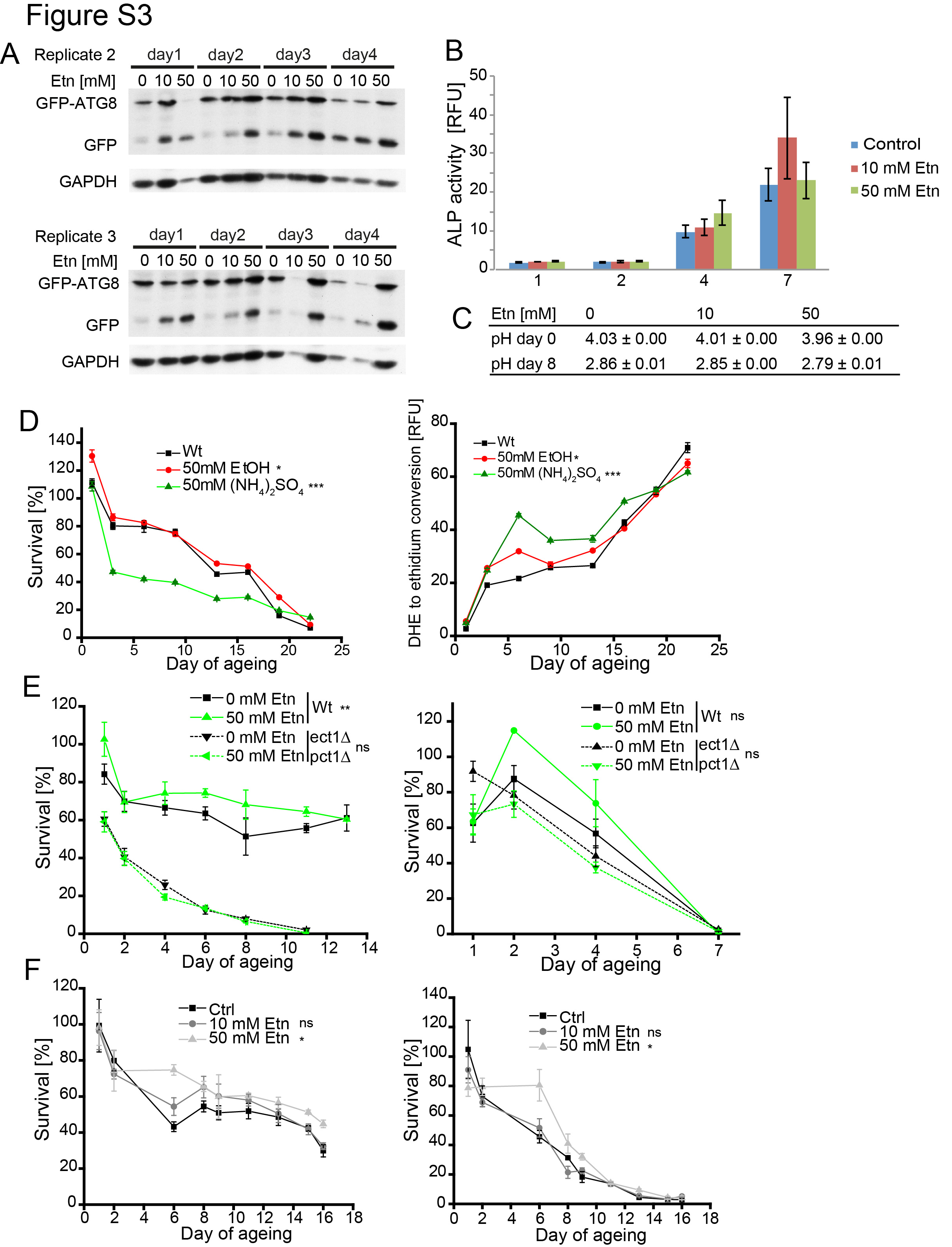


Figure S3. Additional figure related to the ethanolamine supplementation data from Fig. 3. In panel (A) two additional GFP-Atg8 immuno blot replicates from two independent experiments are shown. (B) Measurement of alkaline phosphatase activity in yeast with ethanolamine administration. (C) pH measurement of yeast culture medium at different days of ageing under diverse conditions of ethanolamine administration. (D) Clonogenic survival plating (left panel) and DHE to ethidium conversion (right panel) at different days of ageing with (NH_4_)_2_SO_4_ as a ‘nitrogen-control’ and ethanol as a ‘calorie-control’. (E) Chronological ageing of wildtype (Wt) and ect1∆ pct1∆ DKO in BY4741 (left panel) and BY4742 (right panel) with and without 50 mM ethanolamine (Etn) treatment. (F) Chronological ageing of BY4741 wildtype (left panel) and atg7∆ (right panel) with 0, 10 and 50 mM ethanolamine treatment.


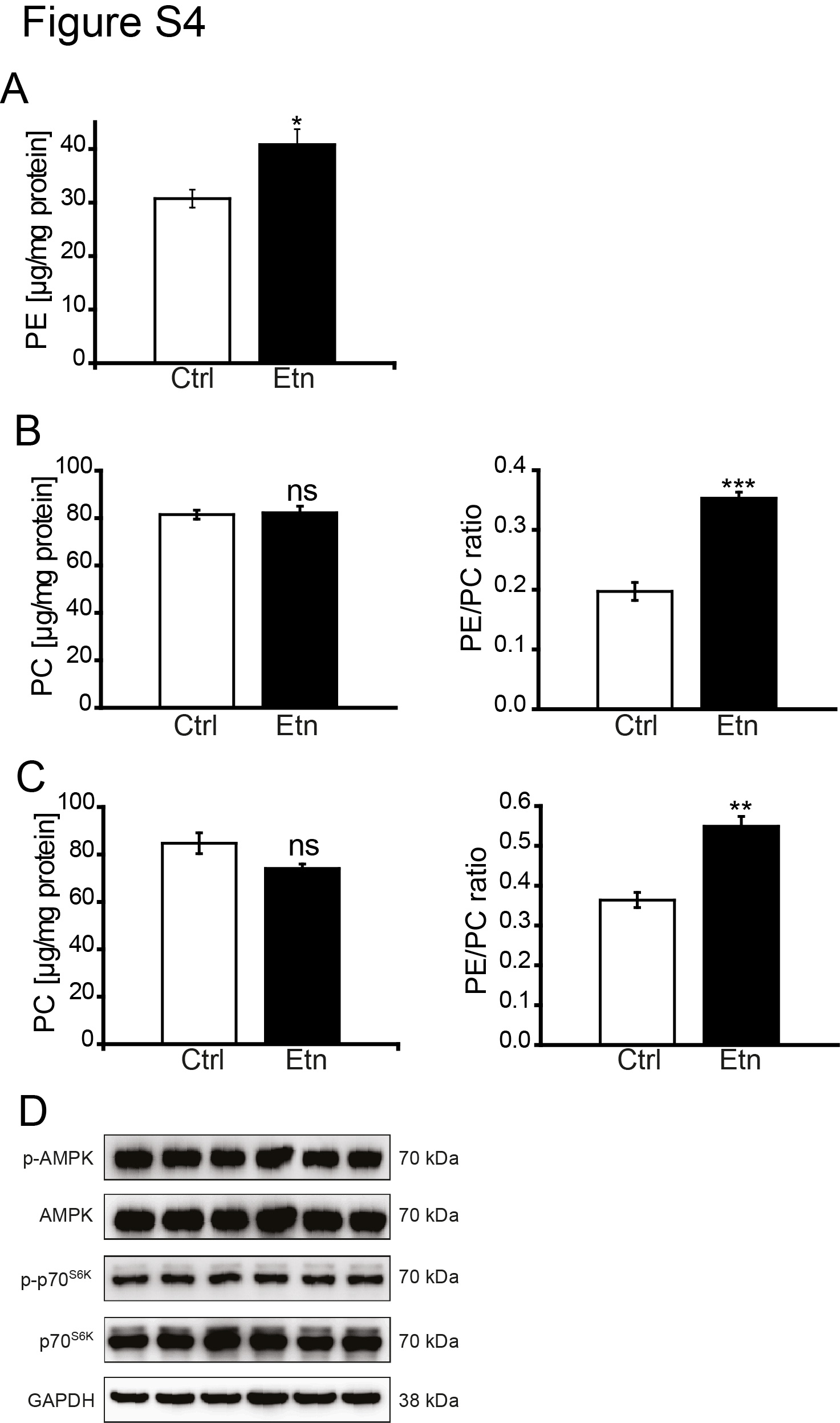


Figure S4. Additional figure related to the ethanolamine administration in mammalian cells from Fig. 4. (A-C) HPLC-ELSD-assisted measurement of total cellular lipid extracts with and without 10 mM ethanolamine (Etn) treatment. Panel (A) depicts the PE quantification in H4 cells, (B) a PC detection (left panel) and PE/PC ratio (right panel) in U2OS and (C) the same measurement in the H4 cell line. Panel (D) depicts a representative immunoblot of AMPK, phosphorylated AMPK, p70^s6k^ and phosphorylated p70^s6k^. Autophagy activation is neither associated with a decrease in mTORC1 activity, as measured by the phosphorylation of p70^s6k^, nor with an activation of AMPK. GAPDH levels are monitored to ensure equal loading.


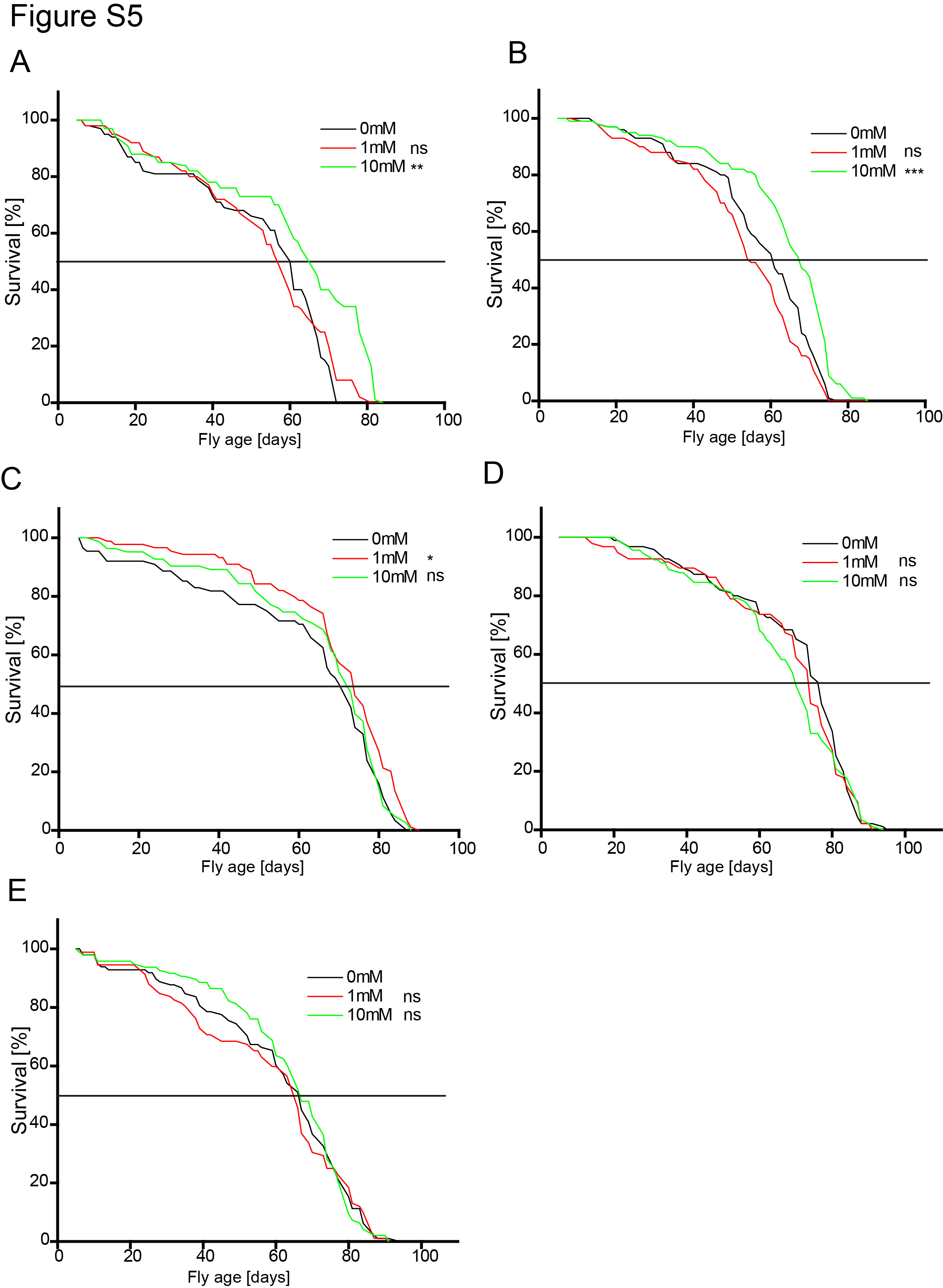


Figure S5. Five independent *Drosophila melanogaster* lifespan analyses with ethanolamine administration. Here each of the five independent female fly lifespan measurement is shown separately (A-E). (A) The mean lifespan of the control population is 52.18 ± 2.52, 52.56 ± 2.44 for flies treated with 1 mM ethanolamine and 60.13 ± 2.71 days for 10 mM treated flies. Wilcoxon comparison of 1 mM treated flies to control flies revealed a χ^2^ of 0 on 1 degree of freedom and p= 0.928, while significant lifespan increase was assessed comparing 10 mM to untreated flies with a χ^2^ of 7.7 on 1 degree of freedom and p= 0.0056. (B) The mean lifespan of the control population is 57.13 ± 1.58, 53.67 ± 1.59 for flies treated with 1 mM ethanolamine and 63.21 ± 1.62 days for 10 mM treated flies. Wilcoxon comparison of 1 mM treated flies to control flies revealed a χ^2^ of 3.6 on 1 degree of freedom and p= 0.0594, while significant lifespan increase was assessed comparing 10 mM to untreated flies with a χ^2^ of 12.5 on 1 degree of freedom and p= 0.000418. (C) The mean lifespan of the control population is 62.34 ± 2.39, 69.67 ± 1.79 for flies treated with 1 mM ethanolamine and 65.86 ± 2.1 days for 10 mM treated flies. Wilcoxon comparison of 1 mM treated flies to control flies revealed a χ^2^ of 5.2 on 1 degree of freedom and p= 0.0225, while significant lifespan increase was assessed comparing 10 mM to untreated flies with a χ^2^ of 0.7 on 1 degree of freedom and p= 0.389. (D) The mean lifespan of the control population is 69.92 ± 1.83, 68.22 ± 1.96 for flies treated with 1 mM ethanolamine and 66.73 ± 1.92 days for 10 mM treated flies. Wilcoxon comparison of 1 mM treated flies to control flies revealed a χ^2^ of 0.6 on 1 degree of freedom and p= 0.426, while significant lifespan increase was assessed comparing 10 mM to untreated flies with a χ^2^ of 2.5 on 1 degree of freedom and p= 0.114. (E) The mean lifespan of the control population is 60.52 ± 2.16, 56.3 ± 2.5 for flies treated with 1 mM ethanolamine and 63.56 ± 1.91 days for 10 mM treated flies. Wilcoxon comparison of 1 mM treated flies to control flies revealed a χ^2^ of 1.2 on 1 degree of freedom and p= 0.275, while significant lifespan increase was assessed comparing 10 mM to untreated flies with a χ^2^ of 0.5 on 1 degree of freedom and p= 0.466.

Supplemental Experimental Procedures

**Generation of Rho^0^ yeast strains**

For abrogation of the mtDNA (Rho^0^), yeast was grown in complete medium containing 10 g/ml ethidium bromide for 3 days, and inoculated in fresh medium each day. The resulting respiratory deficiency was confirmed by a complete lack of growth on obligatory respiratory medium (SC medium with 3% glycerol).

**Additional media**

Glycerol SC medium contained 3% glycerol instead of glucose.

Determination of autophagy rates by assessment of ALP activity

To assess ALP activity, BY4741 wild type or Δach1 cells were transformed with and selected for stable insertion of pTN9 HindIII fragment containing genetically engineered form of PHO8 coding for the Pho8 protein lacking its N-terminal transmembrane domain (Pho8pΔN60) (2). ALP activity was measured similar to Kirisako et al. (3) using 1 μg total protein assessed by BioRad protein assay (BioRad). In order to correct for intrinsic (background) ALP activity, respective strains without pTN9 insertion were processed in parallel and ALP activity subtracted.

Extracellular pH measurement of yeast cultures

The pH of yeast medium was determined using a pH electrode. Measurements were performed at day 0 after ethanolamine administration before inoculation, and at day 8 of chronological ageing. Data are shown as means ± SEM of three independent measurements.

Statistical Analysis

Statistical analyses were calculated in Origin8. For assessment of significance one-way ANOVA followed by Bonferroni post hoc test was performed, except for Figures S1A, B, C and S3D, E, F, which were processed using a two-factor ANOVA with strain and time as independent factors. Data in Fig. S5 were assessed for significant difference by Wilcoxon analysis. Error bars indicate standard error of the mean (SEM) and asterisks in the figures indicate significant differences, *p<0.05, **p<0.01, ***p<0.001.

Supplemental Tables

Table S1. Yeast strains used in this study

| YPR | Name | Genotype | Source/reference |
| --- | --- | --- | --- |
| A01 | Wt | BY4741 MATa his3∆1 leu2∆0 met15∆0 ura3∆0 | EUROSCARF |
| A48 | spo14∆ | BY4741 MATa his3∆1 leu2∆0 met15∆0 ura3∆0 spo14::KanMX4 | EUROSCARF |
| A50 | opi3∆ | BY4741 MATa his3∆1 leu2∆0 met15∆0 ura3∆0 opi3::KanMX4 | EUROSCARF |
| A51 | cpt1∆ | BY4741 MATa his3∆1 leu2∆0 met15∆0 ura3∆0 cpt1::KanMX4 | EUROSCARF |
| A52 | eki1∆ | BY4741 MATa his3∆1 leu2∆0 met15∆0 ura3∆0 eki1::KanMX4 | EUROSCARF |
| A43 | lro1∆ | BY4741 MATa his3∆1 leu2∆0 met15∆0 ura3∆0 lro1::KanMX4 | EUROSCARF |
| A57 | pct1∆ | BY4741 MATa his3∆1 leu2∆0 met15∆0 ura3∆0 pct1::KanMX4 | EUROSCARF |
| A46 | isc1∆ | BY4741 MATa his3∆1 leu2∆0 met15∆0 ura3∆0 isc1::KanMX4 | EUROSCARF |
| A53 | nte1∆ | BY4741 MATa his3∆1 leu2∆0 met15∆0 ura3∆0 nte1::KanMX4 | EUROSCARF |
| A54 | cki1∆ | BY4741 MATa his3∆1 leu2∆0 met15∆0 ura3∆0 cki1::KanMX4 | EUROSCARF |
| A58 | psd1∆ | BY4741 MATa his3∆1 leu2∆0 met15∆0 ura3∆0 psd1::KanMX4 | EUROSCARF |
| A59 | psd2∆ | BY4741 MATa his3∆1 leu2∆0 met15∆0 ura3∆0 psd2::KanMX4 | EUROSCARF |
| D57 | pESC-empty | BY4741 MATa his3∆1 leu2∆0 met15∆0 ura3∆0 pESC-empty | this study |
| D61 | pESC-PSD1 | BY4741 MATa his3∆1 leu2∆0 met15∆0 ura3∆0 pESC-PSD1 | this study |
| J76 | eGFP-ATG8 | BY4741 MATa his3∆1 leu2∆0 met15∆0 ura3∆0 eGFP-ATG8 | Eisenberg et al., Cell Metab, 2014 (4) |
| J57-59 | eGFP-ATG8 psd1∆ | BY4741 MATa his3∆1 leu2∆0 met15∆0 ura3∆0 eGFP-ATG8 psd1::KanMX4 | this study |
| J38-40 | eGFP-ATG8 psd2∆ | BY4741 MATa his3∆1 leu2∆0 met15∆0 ura3∆0 eGFP-ATG8 psd2::KanMX4 | this study |
| J77 | eGFP-ATG8 | BY4742 MATα his3∆1 leu2∆0 lys2∆0 ura3∆0 eGFP-ATG8 | Eisenberg et al., Cell Metab, 2014 (4) |
| J29-31 | eGFP-ATG8 psd1∆ | BY4742 MATα his3∆1 leu2∆0 lys2∆0 ura3∆0 eGFP-ATG8 psd1::KanMX4 | this study |
| J33-35 | eGFP-ATG8 psd2∆ | BY4742 MATα his3∆1 leu2∆0 lys2∆0 ura3∆0 eGFP-ATG8 psd2::KanMX4 | this study |
| L03 | eGFP-ATG8 pESC-empty | BY4742 MATα his3∆1 leu2∆0 lys2∆0 ura3∆0 eGFP-ATG8 pESC-empty | this study |
| L04 | eGFP-ATG8 pESC-PSD1 | BY4742 MATα his3∆1 leu2∆0 lys2∆0 ura3∆0 eGFP-ATG8 pESC-PSD1 | this study |
| J65 | ptn9 pESC-empty | BY4741 MATa his3∆1 leu2∆0 met15∆0 ura3∆0 pho8∆N60 pESC-empty | this study |
| J69 | ptn9 pESC-PSD1 | BY4741 MATa his3∆1 leu2∆0 met15∆0 ura3∆0 pho8∆N60 pESC-PSD1 | this study |
| H33 | ptn9 | BY4741 MATa his3∆1 leu2∆0 met15∆0 ura3∆0 pho8∆N60 | this study |
| L78 | Wt | BY4742 MATα his3∆1 leu2∆0 lys2∆0 ura3∆0 | EUROSCARF |
| J16-18 | psd1∆ | BY4742 MATα his3∆1 leu2∆0 lys2∆0 ura3∆0 psd1::KanMX4 | this study |
| J15 | psd2∆ | BY4742 MATα his3∆1 leu2∆0 lys2∆0 ura3∆0 psd2::KanMX4 | EUROSCARF |
| E9-14 | wt Rho0 | BY4741 MATa his3∆1 leu2∆0 met15∆0 ura3∆0 Rho0 | Rockenfeller et al. 2010 (5) |
| M3-8 | psd1∆ Rho0 | BY4741 MATa his3∆1 leu2∆0 met15∆0 ura3∆0 psd1::KanMX4 Rho0 | this study |
| M11-16 | psd2∆ Rho0 | BY4741 MATa his3∆1 leu2∆0 met15∆0 ura3∆0 psd2::KanMX4 Rho0 | this study |
| F64 | atg7∆ | BY4741 MATa his3∆1 leu2∆0 met15∆0 ura3∆0 atg7::KanMX4 | EUROSCARF |
| J28 | atg7∆ | BY4742 MATα his3∆1 leu2∆0 lys2∆0 ura3∆0 atg7::KanMX4 | EUROSCARF |

**Supplemental References**

1. Horvath SE, Bottinger L, Vogtle FN, Wiedemann N, Meisinger C, Becker T*, et al.* Processing and topology of the yeast mitochondrial phosphatidylserine decarboxylase 1. *J Biol Chem* 2012 Oct 26; **287** (44)**:** 36744-36755.

2. Noda T, Matsuura A, Wada Y, Ohsumi Y. Novel system for monitoring autophagy in the yeast Saccharomyces cerevisiae. *Biochem Biophys Res Commun* 1995 May 5; **210** (1)**:** 126-132.

3. Kirisako T, Baba M, Ishihara N, Miyazawa K, Ohsumi M, Yoshimori T*, et al.* Formation process of autophagosome is traced with Apg8/Aut7p in yeast. *J Cell Biol* 1999 Oct 18; **147** (2)**:** 435-446.

4. Eisenberg T, Schroeder S, Andryushkova A, Pendl T, Kuttner V, Bhukel A*, et al.* Nucleocytosolic depletion of the energy metabolite acetyl-coenzyme a stimulates autophagy and prolongs lifespan. *Cell Metab* 2014 Mar 4; **19** (3)**:** 431-444.

5. Rockenfeller P, Ring J, Muschett V, Beranek A, Buettner S, Carmona-Gutierrez D*, et al.* Fatty acids trigger mitochondrion-dependent necrosis. *Cell Cycle* 2010 Jul 15; **9** (14)**:** 2836-2842.
